# Translating a stress management intervention for rural Latina breast cancer survivors: The *Nuevo Amanecer-II*

**DOI:** 10.1371/journal.pone.0224068

**Published:** 2019-10-16

**Authors:** Jasmine Santoyo-Olsson, Anita L. Stewart, Cathy Samayoa, Helen Palomino, Aday Urias, Nayeli Gonzalez, Alma Torres-Nguyen, LaVerne Coleman, Cristian Escalera, Vicken Y. Totten, Carmen Ortiz, Anna Maria Nápoles

**Affiliations:** 1 Division of General Internal Medicine, Department of Medicine, University of California San Francisco, San Francisco, California, United States of America; 2 Center for Aging in Diverse Communities, University of California San Francisco, San Francisco, California, United States of America; 3 Institute for Health and Aging, University of California San Francisco, San Francisco, California, United States of America; 4 Health Equity Research Lab, Department of Biology, San Francisco State University, San Francisco, California, United States of America; 5 Cancer Resource Center of the Desert, El Centro, California, United States of America; 6 Kaweah Delta Health Care District, Visalia, California, United States of America; 7 WomenCARE/Entre Nosotras, Family Service Agency of the Central Coast, Soquel, California, United States of America; 8 National Institute on Minority Health and Health Disparities, National Institutes of Health, Bethesda, Maryland, United States of America; 9 Círculo de Vida Cancer Support and Resource Center, San Francisco, California, United States of America; Fordham University, UNITED STATES

## Abstract

**Objectives:**

Adapt a cognitive-behavioral stress management program (*Nuevo Amanecer* or *NA*) to be generalizable to rural, low literacy Spanish-speaking Latinas with breast cancer survivors at all phases of survivorship.

**Methods:**

Apply the Transcreation Framework, a community-engaged translational model, to develop the adapted program (*Nuevo Amanecer* or *NA-II*), design a randomized controlled trial for community settings, identify recruiters and interventionists, and recruit participants into the trial.

**Results:**

Adaptations included expanding the program from eight to ten weeks, simplifying materials, and increasing skills practice. We added stress management videos, healthy lifestyles information, and survivorship information. Interventionists were trained Latina breast cancer survivors. All core components of *NA* were retained in *NA-II* including managing the impact of cancer, information on breast cancer and its treatment, finding cancer information, getting support, managing thoughts, stress management techniques, and setting goals. Participants receive a program manual. Each session includes a review of that week’s content using the manual, practicing a stress-management skill, setting a specific goal, and reviewing videos. Spanish-speaking Latinas with non-metastatic breast cancer were recruited by community recruiters. Of 231 women approached, 24% refused, 10% were ineligible, and 153 (66%) were randomized to the intervention or a wait-list control group. The sample was vulnerable: 69% had < high school education, more than half had only Medicaid or no insurance, 91% was foreign born, and 48% reported financial hardship in the past year.

**Conclusions:**

Applying the Transcreation Framework to engage stakeholders in designing community-based RCTs enhanced congruence with community contexts and recruitment of this vulnerable population.

## Introduction

Rural cancer survivors represent a vulnerable and understudied population. Rural cancer patients across the U.S. face challenges receiving cancer care due to poverty and limited access to cancer treatments, cancer support providers, and transportation [1]. Rural residents are more likely to be diagnosed with late-stage cancer and experience higher cancer mortality rates than their urban counterparts [2, 3]. Information is limited about their quality of life following diagnosis [4, 5]. The first national comparison of health status between rural and urban cancer survivors, conducted in 2013, found that rural survivors were more likely to report fair/poor health, psychological distress, and comorbidities, and were at greater risk of poor health for many years after diagnosis than urban survivors [2].

Two systematic reviews found that rural breast cancer survivors have a greater desire for information about their cancer and less access to psychosocial supportive resources than their urban counterparts [6, 7]. Barriers to cancer supportive services among rural women with breast cancer include distance, lack of transportation, low income, and low literacy [1, 8]. Latina breast cancer survivors in rural settings are an especially high-risk group due to the intersections of poverty, limited services, limited transportation, limited education, legal status issues, limited availability of childcare, and language barriers [2]. Most research on Latina breast cancer survivors focuses on urban Latinas. Urban Latinas were at higher risk of psychosocial and physical sequelae of breast cancer than were White women, reporting higher rates of anxiety, depression, fear of recurrence, fatigue, pain, and worse health-related quality of life [9–12] and shorter disease-free survival [12–14].

Cognitive-behavioral stress management (CBSM) interventions are evidence-based cancer supportive services that can improve health-related quality of life, reduce stress and anxiety [15–20], and may reduce chances of recurrence by decreasing inflammation and improving immune responses [16, 19–21]. Cognitive-behavioral stress management interventions generally provide a short-term, practical, skills-based approach that is highly suitable for populations such as ours that live in highly taxing circumstances.

However, only one study of supportive services delivered in rural settings to cancer patients and no studies among rural Latina breast cancer survivors [22] were found. Thus, there is a need for psychosocial health interventions tailored to meet the specific needs of rural Latina breast cancer survivors.

CBSM programs for cancer patients have been delivered primarily by mental health professionals in large cancer centers. Widespread adoption of these programs could be enhanced if they were translated for delivery in community settings by trained peers, and tailored to meet the needs of more vulnerable, ethnically diverse groups. Utilizing peer cancer survivors for program delivery is a promising avenue for increasing access to stress management services in community settings, [23, 24] especially for Latinas. Latina breast cancer survivors inspire hope in other Latina cancer survivors and provide information on how to talk to their physicians and make better treatment decisions [25–27]. Peer health educators have been trained to work effectively among Spanish-speaking Latinas to promote cancer screening, [28] and mental health [29], but peer-delivered cancer support interventions for Latinas living in rural areas with breast cancer need to be further investigated.

Utilizing this strategy in a prior study, a community-based participatory randomized controlled trial (RCT) was conducted to develop and test a CBSM intervention (*Nuevo Amanecer-A New Dawn*, hereafter referred to as *NA*) for recently diagnosed, urban, Spanish-speaking Latina breast cancer survivors [30–32]. The eight-week *NA* program is a culturally tailored, peer-delivered intervention that teaches cognitive-behavioral coping skills to survivors, to manage stress and emotions. The RCT results indicated that women who received the *NA* program improved in several health-related quality of life domains and decreased breast cancer concerns and depressive and bodily symptoms over a six month period, compared to a wait-list control group [31].

Having demonstrated program efficacy for *NA* among urban Latina breast cancer survivors, the program was adapted to address the needs of rural Latina breast cancer survivors and throughout survivorship (not just for recently diagnosed women). As part of this research program to translate evidence-based interventions into community settings to reach vulnerable populations, we published a novel community-engaged translational model called the “Transcreation Framework” [33]. It describes a community-engaged process of planning, delivering, and evaluating interventions in communities to reduce health disparities. Because the focus is on community fit, extensive adaptations often result in a new transcreated program that resonates with the targeted community while achieving the intended health outcomes.

The purpose of this paper is to describe the process employed, using the Transcreation Framework [33], to adapt and improve the generalizability of *NA* to be appropriate for rural, low literacy Spanish-speaking Latina breast cancer survivors throughout survivorship. Also, we describe methods for working with community partners to design a RCT to test the new program *Nuevo Amanecer-II (NA-II*), and enrollment results.

## Methods

To develop *NA-II* and design and implement the RCT in three rural communities, seven steps were followed from the Transcreation Framework [33]. These are: 1) identify community infrastructure and engage partners, 2) specify theory, 3) identify multiple inputs for new program, 4) design intervention prototype, 5) design study, methods, and measures for community setting, 6) build community capacity for delivery, and 7) deliver transcreated intervention and monitor implementation processes.

## Results

In this section, the results of the seven steps are described. For step 7, we report on recruitment results, and describe the sample and methods for monitoring implementation.

### Step 1: Identify community infrastructure and engage partners

The purpose of Step 1 is to identify settings with sufficient infrastructure and relevant experience to deliver the intervention, and potentially sustain it. The project was jointly directed by an academic (PI) and community-based investigator (Co-PI). The academic PI was a Mexican-American behavioral epidemiologist and Professor at the University of California San Francisco (at the time the study was implemented). The community Co-PI was a Puerto Rican clinical psychologist, breast cancer survivor, and Executive Director of Círculo de Vida Cancer Support and Resource Center. The academic PI had primary responsibility for the scientific aspects of the project and the community Co-PI oversaw training and community interventionists.

#### Rural community partners

During the planning phase, county-level cancer registry data were used and U.S. Census data and maps to identify geographically-defined counties of California that could be study sites. The criteria included: 1) rural (defined as counties whose economy is heavily based on agriculture); 2) large Latino immigrant populations; and 3) sufficient numbers of new breast cancer cases among Latinas. Fifteen counties that met these criteria were identified. Within these 15 counties, online searches for organizations that provided cancer supportive services in Spanish were conducted. Eight counties with a community organization that provided cancer supportive services in Spanish were identified. These eight agencies were called and screened for sufficient infrastructure to deliver and potentially sustain the program, experience providing cancer services to the target population, and established relationships with health care providers who could refer potential participants. After screening the organizations, the organizations were offered the possibility of collaborating on the grant proposal and study (if funded) to three organizations that agreed and participated for the duration of the study. The final organizations (and county in which organization is located) were Cancer Resource Center of the Desert (Imperial county), WomenCARE/Entre Nostras (Santa Cruz county), and Kaweah Delta Health Care District (Tulare county). Cancer Resource Center of the Desert is the only local non-profit community-based organization in the Imperial Valley providing comprehensive cancer and patient navigation to Spanish-speaking clients. WomenCARE is a cancer support program of the Family Service Agency of the Central Coast (a mental health services provider) and Entre Nosotras is the Spanish-language arm of WomenCARE. Kaweah Delta Health Care District is the only local hospital in Visalia that offers comprehensive health care services and it employs community health workers who conduct health education and prevention services in the community. These partners were interested in partnering on the study to obtain training on evidence-based cancer support services, to generate evidence on the effectiveness of community-based services, and to add the program to their repertoire of services offered.

### Step 2: Specify theory

Step 2 is to select a theoretical framework that fit the health priority and context, working with the coalition identified in Step 1 [33]. As in *NA*, Social Cognitive Theory [31, 34] was applied in *NA-II*. All sessions incorporated key Social Cognitive Theory components of self-efficacy (e.g., for accessing cancer information, managing thoughts and activities affecting mood), outcome expectations (e.g., recognizing and restructuring unhelpful thoughts about cancer and the future), self-regulation (e.g., self-monitoring and adapting behaviors and cognitions until goals were met), and interventionists modeling behaviors (e.g., relaxation techniques).

### Step 3. Identify multiple inputs for new program

Step 3 in the framework includes reviewing evidence-based interventions, systematic reviews, evidence-based guidelines, and other evidence (e.g., optimal delivery modes) to inform the intervention. This comprehensive level of review was conducted to develop *NA* [30, 32]. For this study, the focus was on refining and tailoring *NA* for a new setting and population. We describe the three inputs: 1) the original *NA* program, 2) debriefing interviews conducted with *NA* participants and interventionists, and 3) new formative research conducted in the new study settings.

#### *NA* program

The foundation for *NA-II* was the *NA* program designed for Latina breast cancer survivors living in urban settings [26, 30]. *NA* emphasized cognitive-behavioral coping skills training, coaching, and modeling to actively manage stress and emotions. Interventionists were trained peers (called compañeras or companions) who delivered the intervention and provided social support. Core program components included stress management skills training (e.g., deep breathing, guided imagery), cognitive reframing (turning unhelpful thoughts into helpful thoughts), effective communication (with clinicians, family, friends), setting goals, and self-regulation to achieve goals. During program sessions, compañeras modeled these skills, had the participant practice them, and provided positive reinforcement.

#### Debriefing interviews from *NA*

After the *NA* RCT was completed, debriefing interviews were conducted with two compañeras (trained breast cancer survivors who delivered the program) and 10 randomly selected program participants (breast cancer patients). Participant surveys and semi-structured interviews were used to assess perceived benefits, quality, ease of use, usefulness of components, and suggested improvements. Debriefing interview results are described in detail elsewhere [32]. Briefly, the following recommendations were made: 1) increase self-management skills practice (healthy lifestyles, ability to cope with stress); 2) better address limited literacy; 3) simplify medical terminology; and 4) include participants’ families.

#### Formative research in new rural settings

New formative research aimed to identify cultural, practical, organizational, and contextual factors in the new settings, to inform program adaptations. Key informant interviews were conducted with two Latina breast cancer survivors, seven cancer supportive services providers (e.g., patient navigator, medical social worker), and two clinicians in the three rural settings to identify local needs. Key informants were identified by community partners as having experience with the target population. A copy of the *NA* program manual was given to informants to review prior to the interviews. Interviews were conducted by the academic PI, community Co-PI, or Project Director. An open-ended interview guide queried informants on suggestions for improvements, how to simplify activities and program delivery, and how to maximize attendance. Informants were asked specific questions about the program manual and activities (e.g., tracking symptoms, setting goals). Interviews were audiotaped and transcribed in English or Spanish.

Informants suggested expanding the program from eight to 10 sessions for more time to practice cognitive and behavioral skills and provide healthy lifestyles information and survivorship care. Informants also suggested flexibility to deliver the intervention individually, in a group, or combination. Other suggestions were to add audiovisual versions (not just text) of the relaxation exercises (deep breathing, visualization) and breast cancer treatment information, simplify medical terminology, supplement text with diagrams, simplify activity sheets and goal setting for low-literacy participants, and include the participants’ families.

### Step 4: Design intervention prototype

Intervention design involved working with the community partners to synthesize and integrate inputs (identified in Step 3) into a new *Participant Program Manual*. It included assuring fit to the new rural community setting and population by tailoring specific program components.

#### Process of adapting *NA* to create *NA-II*

The aim was to modify *NA* to meet the needs of rural Latina breast cancer survivors, be appropriate for women throughout survivorship (not just in the first year after diagnosis), and improve suitability for low-literacy women. The team consisted of the academic PI, community Co-PI, project director and new community partners. Each aspect of the program was discussed by the team, starting with the original *NA* program and integrating the debriefing and key informant interview results and experience with *NA*. Two experienced bilingual-bicultural researchers, the academic PI and project director, consolidated all responses and annotated applicable program manual pages and/or activities with suggested adaptations.

Once initial program adaptations were made, iterative consultations with community partners from the three rural areas informed further program modifications. The compañeras, who were all breast cancer survivors (six total), also reviewed and provided feedback on the program and manual. Final modifications were made based on feedback received during the compañera training. The *NA-II* program and specific modifications are described here.

#### Intervention format and structure

*NA-II* consists of 10 weekly 90-minute sessions, conducted in-person by a compañera. This format and structure are similar to *NA*. Each weekly session (except the first one) begins with a deep breathing exercise, a session overview, and a brief review of the prior week. A *Participant Program Manual* is provided at the first session and used during subsequent sessions. Referring to the manual, the compañera explains the educational content for that week. Each session includes 2–3 activities to reinforce a new stress management technique or skill. The compañera and participant work together on activity sheets, skills practice, role-playing, and discussion of content. Each session ends with a recap and weekly assignment to practice specific skills.

#### Intervention content

Table 1 presents a summary of the 10 final *NA-II* weekly sessions and information on how each session was modified from *NA* if applicable. Sessions cover a range of topics including managing the impact of cancer, breast cancer and survivorship, finding cancer information, getting support, managing thoughts and mood, stress management techniques, managing activities that affect mood, healthy lifestyle, and goal-setting.

**Table 1:** Content of *Nuevo Amanecer-II* intervention and modifications to the original program.

*Nuevo Amanecer–II* program sessions	Modifications to original *Nuevo Amanecer* program
**Week 1: Managing the impact of cancer**	
**Content:** Common reactions to cancer diagnosis or finishing treatment, signs of depression, how to track symptoms, deep breathing for stress management **Activities:** Rate distress on a “distress thermometer” before and after deep breathing **Weekly Goal:** Practice deep breathing for 5 minutes or more every day **DVD Materials:** Deep breathing **Appendices:** Information about the program for family members	– Added reactions to finishing treatment – Simplified deep breathing practice activity sheet – Removed section on breast cancer diagnostic tests – Added information for family members on supportive behaviors
**Week 2: Breast cancer and survivorship**	
**Content:** Breast cancer (types, stages), types of surgeries/treatment, and survivorship care plan (SCP) (purpose, treatment summary, and follow-up care plan) **Activities:** Questions about my cancer, treatment and follow-up care; My Survivorship Care Plan (a blank SCP was provided so they could document their breast cancer care) **Weekly Goal:** Practice deep breathing **DVD Materials:** Deep breathing; understanding breast cancer, radiotherapy, surgery, reconstruction, lymphedema **Appendices:** Breast cancer and its treatments; factors to consider when choosing treatment; common side-effects and long term treatment effects	– Added Arizona Breast Cancer Resource Guide videos (Spanish and English) explaining breast cancer and its treatments [35]. Used lay language – Added a section on SCP and strategies to help participants complete their SCP – Added an activity sheet on questions to ask about cancer, treatment or follow-up care
**Week 3: Finding the cancer information you need**	
**Content:** Communication skills with health care professionals, asking for a professional medical interpreter, participating in and making treatment decisions, calling the National Cancer Institute Cancer Information Service **Activities:** Playing an active role in my care; calling the Cancer Information Service at 1-800-422-6237 **Weekly Goal:** Practice deep breathing **DVD Materials:** Deep breathing	– Removed excessive detail on cancer treatment team members – Added role playing on asking clinicians questions – Moved obtaining second opinion, types of surgeries, and considerations selecting treatments to appendix so as not to overload participants
**Week 4: Getting the support you need**	
**Content:** Effect of cancer diagnosis on family members/friends, talking about cancer with others, communication skills, dealing with criticism, identifying sources of support, asking for help when needed, faith and prayer **Activities:** Practicing good communication skills; people in my life and the ways they support me **Weekly Goals:** Practice deep breathing; practice communication skills around a sensitive issue and reflect on how it went **DVD Materials:** Deep breathing	– Added more culturally appropriate images to reflect target population; replaced images portraying negative behaviors with positive ones – Simplified activity sheet on practicing good communication skills
**Week 5: Thoughts and mood: part 1**	
**Content:** What are thoughts, how thoughts affect mood, identifying unhelpful and helpful thoughts, a simple way to apply cognitive reframing to change negative thoughts with positive thoughts **Activities:** Unhelpful thoughts that I am having; helpful thoughts that I am having; adding “Yes, But” to unhelpful thoughts **Weekly Goals:** Use “Yes, But” technique to change unhelpful thought to helpful thought **DVD Materials:** Deep breathing	– Introduced simpler method to teach cognitive reframing – Added activity practice sheets to reinforce ‘yes, but’ skills
**Week 6: Thoughts and mood: part 2**	
**Content:** Continuation of changing unhelpful thoughts to helpful thoughts, ways to increase helpful thoughts, using coping statements to feel good **Activities:** Changing unhelpful thoughts to helpful thoughts; coping statements before, during and after stressful situations to help reduce stress; my positive and helpful thoughts cards **Weekly Goals:** Read “Positive and Helpful Thought” cards at least once a day and reflect on how it makes you feel **DVD Materials:** Deep breathing; using positive thinking	– Added several cognitive reframing techniques – Provided additional skills training to change unhelpful thoughts to helpful thoughts
**Week 7: Managing stress**	
**Content:** Basics of managing stress, learning healthier ways to manage stress, techniques for handling stress (avoid, change, adapt situations), more relaxation techniques **Activities:** My stress symptoms **Weekly Goals:** Pick one of the new relaxation techniques we learned about today and try it out during the week **DVD Materials:** Deep breathing; guided imagery; progressive muscle relaxation	– Moved relaxation technique instructions (e.g., guided imagery, progressive muscle relaxation) to manual from appendix – Added audiovisuals to help practice relaxation techniques at home & Added stress symptom identification activity
**Week 8: Setting goals to feel better**	
**Content:** Importance of managing activities, how to increase joyful activities, use of laughter, distraction techniques, goal setting **Activities:** What I do affects how I feel; goal setting **Weekly Goals:** Try to achieve the goal that you set for the week **DVD Materials:** Deep breathing	– Simplified goal-setting activity sheet by reducing the instructions, removing the number of fields, and removing the multiple goals per sheet
**Week 9: Setting goals for a healthy lifestyle**	
**Content:** Living a healthy lifestyle after cancer; exercise (benefits, barriers, why is walking important, how to start and set activity goals), nutrition during and post treatment, maintaining healthy weight, getting plenty of sleep, stop smoking, limit alcohol intake **Activities:** What’s getting in my way; setting goals to be more active **Weekly Goals:** Track the goal you set for yourself using activity tracking sheet **DVD Materials:** Deep breathing	– Added self-management skills on healthy lifestyles – Added information on how nutrition and physical activity can alleviate treatment side effects and decrease risk of recurrence
**Week 10: Program recap and future goals**	
**Content:** Review stress management techniques and how to find cancer information, communicating with family/clinicians, increasing helpful thoughts and activities, and setting goals. Closing ceremony. **Activities:** Setting goals to take care of myself **DVD Materials:** Deep breathing **Appendices:** Resources, e.g., support groups; online and phone support; disability benefits; financial assistance for cancer treatment and medication	– Added a healthy living section – Used simplified goal setting activity sheet – Created site-specific community resource guides

Modifications involved improving stress management and cognitive reframing practice and training. The goal setting was simplified to focus on concrete, realistic activities. “Weekly homework” was renamed as “weekly goal” to be less intimidating. Written materials were supplemented with new stress management videos and videos explaining breast cancer and its treatments in lay terms in Spanish and English. All participants are provided a DVD with all videos. These videos are also accessible on YouTube for those who have access to the internet, if they preferred.

#### Compañera and participant manuals

Delivery of the intervention was standardized through compañera and participant program manuals. The *Participant Program Manual* is organized by the 10 weekly sessions and designed to be referred to during each session and to be used independently. It contains information, visual images, and activities developed for lower literacy individuals, written at approximately a sixth-grade reading level. Also included is a DVD (see above). All materials were rigorously translated into Spanish by four bilingual-bicultural research staff, using standard translation methods with team reconciliation.

The *Compañera Program Manual* corresponds to the participant manual but contains additional instructions on how to explain the content, and prompts for the compañera to ask certain questions to aid participant self-reflection. Compañeras are given tablets preloaded with the same audiovisual materials provided to participants. Compañeras use the tablet during sessions to demonstrate program videos of the skills and exercises, such as meditation with guided imagery. Instructions for delivery of *NA-II* are expanded to allow for individual or group format. Nevertheless, all partnering sites decided to deliver the program individually face-to-face, primarily in participants’ homes.

### Step 5: Design study, methods, and measures for community setting

Step 5 focuses on specialized methodological strategies for evaluating the intervention when the study is being conducted in community settings (as opposed to academic settings) where optimal control of study conditions is not possible.

#### Study design

With the community partners, a six-month RCT was designed to assess the effectiveness of *NA-II* in improving breast cancer-specific quality of life (BrQoL) dimensions [36–38]. This RCT compared the 10-week *NA-II* intervention to a wait-list control group. [39]

The Institutional Review Boards (IRB) at the University of California San Francisco (UCSF) and the Kaweah Delta Health Care District approved the protocol. UCSF was the IRB of record for the other two sites. Written informed consent was obtained from all participants.

#### Participant inclusion/exclusion criteria

The study population consisted of Spanish-speaking Latinas with non-metastatic breast cancer residing in the selected rural counties. By design, inclusion criteria were broad because this was an effectiveness trial conducted in real-world settings. Further, it was aimed to increase generalizability to women throughout survivorship. Thus, inclusion criteria were: 1) diagnosis of Stage 0 to IIIC primary breast cancer, 2) lives in Imperial, Tulare, or Santa Cruz/Monterey counties, 3) speaks primarily Spanish, and 4) self-identifies as Latina. Exclusion criteria were: 1) terminal illness, 2) Stage IV breast cancer (distant metastasis), or 3) plans to move out of the area within 6 months.

#### Screening, recruitment, and enrollment

Two sites, Cancer Resource Center of the Desert and WomenCARE/Entre Nosotras, had well-established relationships with local oncology clinics and hospitals. Recruiters identified potentially eligible women face-to-face or on the phone as they performed their normal outreach and cancer education duties. Recruiters verified eligibility of potential participants through medical records review or medical staff confirmation (nurses, patient navigators or social workers) at the clinical sites. The third site, Kaweah Delta Health Care District, was a public hospital and utilized medical records to draw a sample of outpatients who might be eligible. Kaweah Delta Health Care District mailed potential participants an initial contact letter, a study information sheet, and a prepaid refusal postcard to return to the District. The letter and information sheet were in English and Spanish and written at a 6th grade level. If no refusal postcard was received within two weeks, the recruiter initiated contact with the potential participant.

During the initial contact, recruiters used a bilingual script to confirm the woman’s diagnosis, stage at diagnosis, self-reported ethnicity, and absence of terminal illness. When the contact was by phone, if the woman expressed interest in the study and was eligible, an appointment was scheduled at a convenient time and location. At that appointment, the recruiter described the study and answered questions. If the woman agreed, written informed consent and a breast cancer medical records release form were completed. The baseline interview was conducted, followed by randomization. When the initial contact was in-person, recruiters used the same procedures except that women had the option of completing the interview at that time or scheduling an appointment.

#### Randomization

The individual was the unit of randomization with 1:1 allocation to experimental groups. Randomization was stratified by recruitment site. Prior to initiating recruitment, stratum-specific sequential identification numbers were generated and randomly pre-assigned within blocks of random sizes. After the baseline assessment, the recruiter handed each participant a sealed, opaque envelope preprinted with the next sequential identification number from her stratum that revealed her group assignment. Women were randomized to either the intervention group or wait-list control group. Wait-list control group participants were offered the intervention after the six-month assessment.

#### Assessments

Primary and secondary outcomes were assessed at baseline, three months, and six months. All assessments were completed in Spanish. Participants were compensated $30 per assessment, and $10 at the end of the intervention for completing a brief telephone survey about the program. The baseline assessment was conducted in-person; 3- and 6-month assessments occurred by telephone for practical reasons.

Primary outcomes were the Functional Assessment of Cancer Therapy—Breast (FACT-B) [39] subscales and total score. The FACT-B has been translated into Spanish [40] and assesses breast cancer-specific QoL. The FACT-B comprises five subscales pertaining to four dimensions of well-being (physical, social/family, emotional, functional) plus concerns about breast cancer. Secondary outcomes consisted of four dimensions of psychological distress: depressive symptoms, anxiety, somatization, and perceived stress, and stress management skills and confidence. To measure anxiety and somatization, two scales from the Brief Symptom Inventory (BSI) [41] were used. The Spanish version of the PHQ-8 was used to assess depressive symptoms [42, 43]. The Spanish version of the 10-item Perceived Stress Scale (PSS-10), [44, 45] previously used in the HCHS/SOL Sociocultural Ancillary Study, [46, 47] was used.

We measured women’s ability to practice four of the self-management skills taught in *NA*-II, we used the Measure of Current Status Part A (MOCS-A) [48]. We used a Spanish translation provided by Dr. Frank J. Penedo (personal communication). The MOCS-A comprises four subscales. Awareness of tension measures their ability to be aware of tightness in their body, recognize stressful situations, and notice when their body is becoming tense. Relaxation assessed women’s ability to use muscle relaxation and mental imagery (relaxation techniques) to reduce tension. Assertiveness measures the extent to which women were able to ask for help, support, or assistance when needed and could clearly express their needs. Coping confidence measures their ability to re-examine their thoughts to gain a new perspective, decide how to cope with problems, come up with emotionally balanced thoughts, and choose the best coping responses.

Our target of 140 women was based on a power analyses that assumed 80% power; two-tailed α = .05; 90% retention; Functional Assessment of Cancer Therapy—Breast (FACT-B) [39] subscale outcomes at baseline, three and six months; and intention-to-treat analyses with focus on group-by-time interaction effects (minimum detectable effect of the proposed design as estimated by simulation is *d* = .45).

### Step 6. Build community capacity for delivery

Building capacity focuses on enhancing the community infrastructure identified in Step 1. The methods for building capacity of the participating organizations in order to deliver the program are highlighted here.

#### Participating organizations

The project was funded through the California Breast Cancer Research Program Translational Research Award mechanism. Although the academic partner was awarded the grant, roughly half of the funding was provided to the community Co-PI’s agency (Círculo de Vida) via a subcontract. The community Co-PI’s agency, in turn, managed and disbursed the funds for program implementation to the community agencies. Each partner site received an organizational fee of $45,000 from the community Co-PI to offset agency costs to pay site recruiters, compañeras, and overhead. It gave them ownership of their own budget, thus enabling each site to identify what worked best within their organization. Each site decided on the pay rate of their recruiters and compañeras. In some cases, recruiters were paid on an hourly basis, and in other cases, the funds were used to pay part of their salary as this project was added to their job description. The Co-PI’s organization reimbursed recruiters and compañeras for mileage ($0.50 per mile) incurred to enroll or meet with participants for program delivery.

To build organizational capacity, through monthly conference calls, all partners were involved in most aspects of the research, including developing the grant proposal, adapting the intervention, designing recruitment materials and strategies, and study implementation and evaluation. Conference calls were an important forum for reviewing recruitment results, brainstorming ways to improve recruitment and outreach, sharing rewards and challenges, and discussing program sustainability and dissemination. Calls continued on a monthly basis until the final assessments were completed, after which they continued on a quarterly basis for the remainder of the study.

#### Community-based peers to recruit and enroll participants

Each partner site identified a recruiter applicant pool. Recruiters had to be bilingual and have prior health education or outreach experience and access to Latina breast cancer patients through their community activities or contacts. The academic PI and project director screened candidates using a structured interview protocol that assessed their interpersonal communication skills, relevant experience, and outreach skills.

A four-hour training session conducted by the academic PI and project director prepared recruiters to recruit, enroll, randomize, and conduct baseline assessments. The training included a PowerPoint presentation on the study, review of enrollment and tracking forms, review of the baseline survey, and role modeling of interview skills and study procedures. Each recruiter was provided with a recruiter manual that included information on the study protocol, recruiter and compañera roles, and detailed procedures for tracking, identifying, screening for eligibility, obtaining informed consent, conducting the baseline interviews, and randomization. Two recruiters completed training in each site.

Community recruiters also completed human subjects protection training, either on the Collaborative Institutional Training Initiative website or through in-person training by the academic PI and project director using the Community Involvement in Research Training (CIRTification) [49] developed by The University of Illinois at Chicago Center for Clinical and Translational Science. CIRTification is a human research protection training program designed specifically for community research partners who do not have prior experience with research. The training was essential for a few recruiters who were not comfortable receiving computer-based training. Use of CIRTification was approved by the study’s two institutional review boards (academic and hospital site IRBs). Recruiters were compensated for completing the training. The academic project director provided on-going supervision.

#### Community-based interventionists (compañeras)

As in *NA*, peers delivered the intervention. Compañeras were breast cancer survivors and served as role models to inspire hope. The community partners identified potential candidates. Compañera requirements were being bilingual or Spanish-monolingual, a Latina breast cancer survivor, and at least three years post diagnosis with no recurrence. The community-based Co-PI (a clinical psychologist) interviewed potential candidates using a structured interview protocol. Compañeras needed to display warmth and compassion to create a strong sense of familismo (familial interconnectedness) and confianza (trust). Sensitivity to spirituality was important as Latinas often use faith as a source of support and acceptance. Women had to demonstrate having processed their experiences being diagnosed with breast cancer and effective coping and communication skills.

Compañeras participated in three eight-hour training sessions conducted in Spanish by the academic PI, project director, community Co-PI, and the community Co-PI’s clinical director. The training included didactic presentations, hands-on review of the program manuals, and active practice of the skills training and other activities, with feedback from trainers. Training materials include a *Compañera Training Manual* in addition to the *Compañera Program Manual*. The training started with a PowerPoint presentation that covered psychosocial issues among Latinas with breast cancer living in rural communities, theoretical basis of the program, results of the *NA* study, protocol, randomization, role of recruiters and compañeras, overview of compañera manual, logistics of program delivery, and how to collect process evaluation and fidelity indicators. The training continued with detailed hands-on review of the *Compañera Program Manual*, role playing, skills demonstration and practice. The manual included step-by-step instructions (e.g., purpose of the activity, questions to ask) and materials (e.g., activity sheets) for the 10 weekly sessions. Compañeras were compensated for attending training and they received a certificate of completion.

Compañeras were trained to follow the manual, but encouraged to make it client-centered such that they could reinforce sections when the client verbalized or demonstrated a need for more practice, or omit sections that were not relevant (e.g., communicating about cancer with young children if they had none). They were instructed to follow the manual content, but make the session conversational. The community Co-PI accompanied the compañeras on their initial intervention sessions until she was satisfied that they were delivering the intervention as intended; the Co-PI also conducted two additional in-person observations with each of the compañeras to determine the extent to which the program was presented as intended. These encounters were audio-recorded and the Co-PI rated the quality of delivery of the program using a structured form. The audio-recordings of the encounters were also rated independently by the Project Director using the structured form. In addition, the Co-PI provided on-going supervision via telephone.

### Step 7: Deliver transcreated intervention and monitor implementation processes

Step 7 involves implementing the intervention and monitoring implementation processes. A description of the first part of this step–recruitment results and the sample characteristics are provided, as well as our methods for monitoring implementation.

#### Screening and recruitment results

The study recruitment period was from September 2016 through March 2018. The enrollment goal of 140 women was exceeded. In total, 231 women were approached, 153 enrolled and were randomized, 24 were ineligible due to language (e.g., did not speak Spanish), and 54 refused, with an overall recruitment rate of 66% (153/231). Of those that refused, 28 were not interested, 19 were too busy/no time, and 7 were moving out of the study area.

#### Sample characteristics

One hundred fifty-three Spanish-speaking Latina breast cancer survivors were enrolled (Table 2). The mean age was 54.8 years (SD 10.5; range 28–88), 80% had a high school education or less, 66% were married, and most were limited English-speaking. Of these, 91% were foreign born. Financial hardship in the past year was reported by 48% of participants, and 18% reported being employed (full/part time). More than half (59%) reported having only Medicaid insurance. Poor/fair health was reported by almost half of participants (46%).

**Table 2:** Baseline characteristics of rural Spanish-speaking Latina breast cancer survivors participating in the *Nuevo Amanecer-II* study, California, September 2016 to March 2018.

Characteristic	Total Sample N = 153
**Age in years (range 28–88), M (SD)**	54.8 (10.4)
**Educational attainment, n (%)**	
Elementary (6 years) or less	60 (39.2)
More than elementary to so some high school	46 (30.1)
High school graduate	17 (11.1)
More than high school	29 (19.0)
Missing	1 (0.6)
**Health insurance, n (%)**	
Private	24 (15.7)
MediCal (Medicaid)	88 (57.5)
Medicare + (Medicaid or private)	38 (24.8)
No insurance	1 (0.7)
Don’t know	2 (1.3)
**Employment status, n (%)**	
Working full or part-time	28 (18.3)
Unemployed	9 (5.9)
Homemaker, caregiver, student	63 (41.2)
Retired	14 (9.2)
Unable to work due to disability	39 (25.5)
**Any financial hardship in past year, n (%)**	74 (48.4)
**Country of birth, n (%)**	
USA	14 (9.2)
Mexico	138 (90.2)
El Salvador	1 (0.7)
**Poor or fair self-rated health, n (%)**	70 (45.8)
**Poor or fair self-rated mental health, n (%)**	54 (35.3)
**Time since most recent diagnosis (range 0–17), M (SD)**	2.5 (3.2)
**Type of breast cancer, n (%)**	
Ductal carcinoma *in situ*	15 (9.8)
Invasive	117 (76.5)
Inflammatory	15 (9.8)
Missing	6 (3.9)
**Stage, n (%)**	
0	8 (5.2)
1	45 (29.4)
2	55 (35.9)
3	27 (17.6)
Missing	18 (11.8)
**Surgery, n (%)**	
Lumpectomy	76 (49.7)
Mastectomy	72 (47.1)
None	1 (0.7)
Missing	4 (2.6)
**Treatment, n (%)**	
Both radiation and chemotherapy	91 (59.5)
Only radiation	28 (18.3)
Only chemotherapy	22 (14.4)
No treatment	10 (6.5)
Missing	2 (1.3)

Of 153 women randomized, years since most recent diagnosis was 2.5 years (SD 3.2; range 0–17); eight were recurrences. Of these, 10% were diagnosed with ductal carcinoma *in situ*, 76% with invasive breast cancer, 10% with inflammatory breast cancer, and 4% had missing information or medical records could not be obtained. Regarding surgery type, 50% had a lumpectomy, 47% had a mastectomy, 1% had no surgery, and 3% were missing. Regarding treatment, 60% received both radiation and chemotherapy, 18% had radiation only, 14% had chemotherapy only, 7% had no treatment, and 1% was missing.

#### Monitoring implementation processes

To assess participant adherence to the program, compañeras completed a structured program tracking form after each of 10 sessions. Data included: logistics (session date, location, duration, and round trip travel time and mileage), whether the participant had completed the homework for that week (yes or no), whether the participant reported difficulty in doing the homework (yes or no) and if so, the type of difficulty (open-ended). For sessions 2–10, the compañera assessed whether the participant was able to demonstrate the skill covered in the prior session (yes or no). The tracking form was also used to document reasons why women dropped out at any point. This information was collected for women in the intervention group and those in the control group who elected to receive the program after the wait period. Program adherence was defined as having completed at least 7 of the 10 sessions.

To assess fidelity, two intervention sessions for each compañera were selected for observation based on convenience. The observer was either the community Co-PI or the community Co-PI’s clinical director. Using structured rating scales (1 = not at all, 2 = a fair amount, 3 = a great deal, 4 = constantly) the observer rated compañera's compliance with six program components: the extent to which they followed the manual for that session, explained concepts in language the participant understood, checked that the participant understood the material, spoke in a caring/supportive way, modeled the skills, and provided feedback to participants for efforts to practice the skills. Seven additional questions rated the extent to which the compañera encouraged participants to practice seven skills (cognitive reframing, good communication, seeking cancer information, practicing stress management, asking for help, increasing helpful activities, and goal setting). Sessions were audiotaped and reviewed independently by two people (project director and a trained health education intern).

## Discussion

For this project, the Transcreation Framework [33] was applied to guide the processes of engaging community partners, building capacity, designing, implementing and evaluating a stress management intervention for rural Latina breast cancer survivors. Following the steps of the framework to engage partners, needed program adaptations were identified. The framework helped us design a rigorous yet practical evaluation of this behavioral intervention in underserved community settings where research resources were limited. Finally, engaging the community partners throughout allowed us to exceed our recruitment goals and facilitated successful implementation of the RCT and program. This RCT is the first to test a CBSM intervention tailored specifically for low-literacy Spanish-speaking Latina breast cancer patients living in rural areas. We demonstrated that such cancer support programs can be extended beyond large cancer centers and urban settings, and implemented in rural community settings using community interventionists who are culturally and experientially congruent with the target population.

In this second iteration of the *NA* program, the content and delivery format were refined based on input from survivors, clinicians, and advocates. Major modifications included adding sessions on healthy lifestyles and further practice of cognitive reframing skills. Audiovisual materials were developed to supplement text information, further simplified medical terminology and activity sheets, and added instructions for delivering the program in a group format. Finally, content relevant for longer term survivors was added, e.g., survivorship care plans and symptom management. Without the involvement of community experts, the need for many of these adaptations might have been missed [50].

A fundamental feature of the Transcreation Framework is community capacity building to deliver behavioral interventions. In this study, partners increased their capacity to deliver evidence-based cancer supportive services and participate in rigorous behavioral research, including RCTs. As a result of their involvement in this study, two of the community organizations have indicated that they are working now with other academic or public entities on planning of future studies and adaptation of the program for other conditions. One of the organizations has incorporated *NA* materials into their support groups. As a result of the *NA* projects, Círculo de Vida Cancer Support and Resource Center was awarded a grant from The Foundation for Hope & Grace to extend the program to three additional rural communities where twenty-one Latina breast cancer survivors were trained and provided the 10-week program to 120 Latinas living with breast cancer.

The three community sites in the study varied in nature, indicating that a variety of types of community organizations can successfully engage in research to improve psychosocial services for vulnerable cancer populations. In this study, one was a public health care system, one was a community-based mental health services provider, and one was a community-based cancer supportive care provider. Thus, researchers can consider a broad spectrum of community organizations and service providers as potential partners when undertaking community-engaged research.

There were study limitations. First, the majority of the sample was of Mexican origin and may not reflect experiences of Spanish-speaking Latinas of other national origin groups. Second, the sample was limited to Latinas living in rural California and may not be generalizable to the Latina cancer survivors living in other regions.

Two promising directions for future research are to address the roles of caregivers and family members and how variations in intervention intensity and delivery modes affect outcomes. In the formative research in both urban and rural settings, key informants suggested that family members be included in the intervention. Although there were insufficient resources to extend the program to family members, a handout was designed for family members that described the content of the program, what changes they might notice in their family member, and how to support her through the program. Development and testing of family-centered and dyadic interventions are future directions for research. Related to program intensity and delivery mode, the only other stress management intervention in the literature that targeted rural cancer patients (not Latinas and not breast cancer-specific) found that women who participated in a 4-week group-based videoconference program felt disconnected from others and desired a longer program [22]. Additional research on optimal modes and intensity of interventions are warranted.

The utility of the Transcreation Framework stems from engaging the community throughout, building community capacity, and conducting a rigorous evaluation of the behavioral intervention in community settings. These implementation and translation strategies could be powerful tools for testing the transportability of effective behavioral interventions among underserved vulnerable populations [50]. Increased program adoption could potentially benefit the most vulnerable cancer survivors.

## References

[1] Charlton M, Schlichting J, Chioreso C, Ward M, Vikas P. Challenges of rural cancer care in the United States. Oncology (Williston Park). 2015;29:633–40. 26384798

[2] Weaver KE, Geiger AM, Lu L, Case LD. Rural-urban disparities in health status among US cancer survivors. Cancer. 2013;119:1050–7. 10.1002/cncr.27840 23096263 PMC3679645

[3] Henley SJ, Anderson RN, Thomas CC, Massetti GM, Peaker B, Richardson LC. Invasive cancer incidence, 2004–2013, and deaths, 2006–2015, in nonmetropolitan and metropolitan counties—United States. MMWR Surveill Summ. 2017;66:1–13. 10.15585/mmwr.ss6614a1 28683054 PMC5879727

[4] Reid-Arndt SA, Cox CR. Does rurality affect quality of life following treatment for breast cancer? J Rural Health. 2010;26:402–5. 10.1111/j.1748-0361.2010.00295.x 21029176 PMC2967464

[5] Pedro LW. Quality of life for long-term survivors of cancer: influencing variables. Cancer Nurs. 2001;24:1–11. 11219417 10.1097/00002820-200102000-00001

[6] Bettencourt BA, Schlegel RJ, Talley AE, Molix LA. The breast cancer experience of rural women: a literature review. Psychooncology. 2007;16:875–87. 10.1002/pon.1235 17611958

[7] Butow PN, Phillips F, Schweder J, White K, Underhill C, Goldstein D. Psychosocial well-being and supportive care needs of cancer patients living in urban and rural/regional areas: a systematic review. Support Care Cancer. 2012;20:1–22. 10.1007/s00520-011-1270-1 21956760

[8] Angell KL, Kreshka MA, McCoy R, Donnelly P, Turner-Cobb JM, Graddy K, et al Psychosocial intervention for rural women with breast cancer: the Sierra-Stanford partnership. J Gen Intern Med. 2003;18:499–507. 10.1046/j.1525-1497.2003.20316.x 12848832 PMC1494883

[9] Graves KD, Jensen RE, Canar J, Perret-Gentil M, Leventhal KG, Gonzalez F, et al Through the lens of culture: quality of life among Latina breast cancer survivors. Breast Cancer Res Treat. 2012;136:603–13. 10.1007/s10549-012-2291-2 23085764 PMC3547364

[10] Yanez B, Thompson EH, Stanton AL. Quality of life among Latina breast cancer patients: a systematic review of the literature. J Cancer Surviv. 2011;5:191–207. 10.1007/s11764-011-0171-0 21274649 PMC3096762

[11] Costas-Muniz R, Hunter-Hernandez M, Garduno-Ortega O, Morales-Cruz J, Gany F. Ethnic differences in psychosocial service use among non-Latina white and Latina breast cancer survivors. J Psychosoc Oncol. 2017;35:424–37. 10.1080/07347332.2017.1310167 28332946 PMC5647778

[12] Apollo AJ, Crew KD, Campbell J, Greenlee H, Jacobson JS, Grann V, et al High rates of psychosocial stress among Hispanic breast cancer survivors. J Clin Oncol. 2007;Vol 25 (Jun 20 Supplement)(No. 18S):9113 {cited 2019 Apr 19}. Available from: https://ascopubs.org/doi/abs/10.1200/jco.2007.25.18_suppl.9113 Subscription required

[13] Bowen DJ, Alfano CM, McGregor BA, Kuniyuki A, Bernstein L, Meeske K, et al Possible socioeconomic and ethnic disparities in quality of life in a cohort of breast cancer survivors. Breast Cancer Res Treat. 2007;106:85–95. 10.1007/s10549-006-9479-2 17260096 PMC2999962

[14] Mukhtar RA, Moore AP, Nseyo O, Baehner FL, Au A, Moore DH, et al Elevated PCNA+ tumor-associated macrophages in breast cancer are associated with early recurrence and non-Caucasian ethnicity. Breast Cancer Res Treat. 2011;130:635–44. 10.1007/s10549-011-1646-4 21717106

[15] Antoni MH, Wimberly SR, Lechner SC, Kazi A, Sifre T, Urcuyo KR, et al Reduction of cancer-specific thought intrusions and anxiety symptoms with a stress management intervention among women undergoing treatment for breast cancer. Am J Psychiatry. 2006;163:1791–7. 10.1176/ajp.2006.163.10.1791 17012691 PMC5756627

[16] Carlson LE, Speca M, Patel KD, Goodey E. Mindfulness-based stress reduction in relation to quality of life, mood, symptoms of stress and levels of cortisol, dehydroepiandrosterone sulfate (DHEAS) and melatonin in breast and prostate cancer outpatients. Psychoneuroendocrinology. 2004;29:448–74. 10.1016/s0306-4530(03)00054-4 14749092

[17] Henderson VP, Clemow L, Massion AO, Hurley TG, Druker S, Hebert JR. The effects of mindfulness-based stress reduction on psychosocial outcomes and quality of life in early-stage breast cancer patients: a randomized trial. Breast Cancer Res Treat. 2012;131:99–109. 10.1007/s10549-011-1738-1 21901389 PMC3784652

[18] Loprinzi CE, Prasad K, Schroeder DR, Sood A. Stress management and resilience training (SMART) program to decrease stress and enhance resilience among breast cancer survivors: a pilot randomized clinical trial. Clin Breast Cancer. 2011;11:364–8. 10.1016/j.clbc.2011.06.008 21831722

[19] Savard J, Simard S, Ivers H, Morin CM. Randomized study on the efficacy of cognitive-behavioral therapy for insomnia secondary to breast cancer, part II: immunologic effects. J Clin Oncol. 2005;23:6097–106. 10.1200/JCO.2005.12.513 16135476

[20] Witek-Janusek L, Albuquerque K, Chroniak KR, Chroniak C, Durazo-Arvizu R, Mathews HL. Effect of mindfulness based stress reduction on immune function, quality of life and coping in women newly diagnosed with early stage breast cancer. Brain Behav Immun. 2008;22:969–81. 10.1016/j.bbi.2008.01.012 18359186 PMC2586059

[21] Antoni MH, Lutgendorf SK, Cole SW, Dhabhar FS, Sephton SE, McDonald PG, et al The influence of bio-behavioural factors on tumour biology: pathways and mechanisms. Nat Rev Cancer. 2006;6:240–8. 10.1038/nrc1820 16498446 PMC3146042

[22] Zhou ES, Partridge AH, Blackmon JE, Morgan E, Recklitis CJ. A pilot videoconference group stress management program in cancer survivors: lessons learned. Rural Remote Health. 2016;16:3863 27303955

[23] Giese-Davis J, Bliss-Isberg C, Carson K, Star P, Donaghy J, Cordova MJ, et al The effect of peer counseling on quality of life following diagnosis of breast cancer: an observational study. Psychooncology. 2006;15:1014–22. 10.1002/pon.1037 16555366

[24] Schover LR, Jenkins R, Sui D, Adams JH, Marion MS, Jackson KE. Randomized trial of peer counseling on reproductive health in African American breast cancer survivors. J Clin Oncol. 2006;24:1620–6. 10.1200/JCO.2005.04.7159 16575013

[25] Banas JR, Victorson D, Gutierrez S, Cordero E, Guitleman J, Haas N. Developing a peer-to-peer mHealth application to connect Hispanic cancer patients. J Cancer Educ. 2017;32:158–65. 10.1007/s13187-016-1066-6 27364905 PMC7770498

[26] Napoles-Springer AM, Ortiz C, O'Brien H, Diaz-Mendez M. Developing a culturally competent peer support intervention for Spanish-speaking Latinas with breast cancer. J Immigr Minor Health. 2009;11:268–80. 10.1007/s10903-008-9128-4 18340533 PMC3832434

[27] Sheppard VB, Figueiredo M, Canar J, Goodman M, Caicedo L, Kaufman A, et al Latina a Latina: developing a breast cancer decision support intervention. Psychooncology. 2008;17:383–91. 10.1002/pon.1239 17628037

[28] Allen JD, Perez JE, Tom L, Leyva B, Diaz D, Idali Torres M. A pilot test of a church-based intervention to promote multiple cancer-screening behaviors among Latinas. J Cancer Educ. 2014;29:136–43. 10.1007/s13187-013-0560-3 24132541 PMC4089980

[29] Corrigan PW, Torres A, Lara JL, Sheehan L, Larson JE. The healthcare needs of Latinos with serious mental illness and the potential of peer navigators. Adm Policy Ment Health. 2017;44:547–57. 10.1007/s10488-016-0737-2 27236458 PMC5997453

[30] Napoles AM, Santoyo-Olsson J, Ortiz C, Gregorich S, Lee HE, Duron Y, et al Randomized controlled trial of Nuevo Amanecer: a peer-delivered stress management intervention for Spanish-speaking Latinas with breast cancer. Clin Trials. 2014;11:230–8. 10.1177/1740774514521906 24577971 PMC3972263

[31] Napoles AM, Ortiz C, Santoyo-Olsson J, Stewart AL, Gregorich S, Lee HE, et al Nuevo Amanecer: results of a randomized controlled trial of a community-based, peer-delivered stress management intervention to improve quality of life in latinas with breast cancer. Am J Public Health. 2015;105 Suppl 3:e55–63. 10.2105/AJPH.2015.302598 25905829 PMC4455521

[32] Napoles AM, Santoyo-Olsson J, Stewart AL, Ortiz C, Garcia-Jimenez M. Evaluating the implementation of a translational peer-delivered stress management program for Spanish-speaking Latina breast cancer survivors. J Cancer Educ. 2018;33:875–84. 10.1007/s13187-017-1202-y 28275966 PMC5591043

[33] Napoles AM, Stewart AL. Transcreation: an implementation science framework for community-engaged behavioral interventions to reduce health disparities. BMC Health Serv Res. 2018;18:710 10.1186/s12913-018-3521-z 30208873 PMC6134771

[34] Bandura A. Social foundations of thought and action: a social cognitive theory. Englewood Cliffs, NJ: Prentice Hall; 1986.

[35] Arizona Breast Cancer Resource Guide. Understanding breast cancer—Arizona Breast Cancer Resource Guide (ABCRG) {online videos}; 2014 {cited 2019 Apr 19}. Available from: https://youtu.be/LeWZoyNX-60

[36] Graves KD, Carter CL, Anderson ES, Winett RA. Quality of life pilot intervention for breast cancer patients: use of social cognitive theory. Palliat Support Care. 2003;1:121–34. 16594274 10.1017/s1478951503030268

[37] Helgeson VS, Cohen S., Schulz R. & Yasko J. Group support interventions for women with breast cancer: who benefits from what? Health Psychol. 2000;19:107–14 10762094 10.1037//0278-6133.19.2.107

[38] Richardson MA, Post-White J, Grimm EA, Moye LA, Singletary SE, Justice B. Coping, life attitudes, and immune responses to imagery and group support after breast cancer treatment. Altern Ther Health Med. 1997;3:62–70. 9287446

[39] Brady MJ, Cella DF, Mo F, Bonomi AE, Tulsky DS, Lloyd SR, et al Reliability and validity of the Functional Assessment of Cancer Therapy-Breast quality-of-life instrument. J Clin Oncol. 1997;15:974–86. 10.1200/JCO.1997.15.3.974 9060536

[40] Cella D, Hernandez L, Bonomi AE, Corona M, Vaquero M, Shiomoto G, et al Spanish language translation and initial validation of the functional assessment of cancer therapy quality-of-life instrument. Med Care. 1998;36:1407–18. 10.1097/00005650-199809000-00012 9749663

[41] Derogatis LR, Melisaratos N. The Brief Symptom Inventory: an introductory report. Psychol Med. 1983;13:595–605. 6622612

[42] Kroenke K, Strine TW, Spitzer RL, Williams JB, Berry JT, Mokdad AH. The PHQ-8 as a measure of current depression in the general population. J Affect Disord. 2009;114:163–73. 10.1016/j.jad.2008.06.026 18752852

[43] Merz EL, Malcarne VL, Roesch SC, Riley N, Sadler GR. A multigroup confirmatory factor analysis of the Patient Health Questionnaire-9 among English- and Spanish-speaking Latinas. Cultur Divers Ethnic Minor Psychol. 2011;17:309–16. 10.1037/a0023883 21787063 PMC4210271

[44] Cohen S, Kamarck T, Mermelstein R. A global measure of perceived stress. J Health Soc Behav. 1983;24:385–96. 6668417

[45] Cohen S, Williamson G. Perceived stress in a probability sample of the United States In: Sparapan W, Oskamp S, editors. The social psychology of health. Newbury Park, CA: Sage Publishers; 1988

[46] Perera MJ, Brintz CE, Birnbaum-Weitzman O, Penedo FJ, Gallo LC, Gonzalez P, et al Factor structure of the Perceived Stress Scale-10 (PSS) across English and Spanish language responders in the HCHS/SOL Sociocultural Ancillary Study. Psychol Assess. 2017;29:320–8. 10.1037/pas0000336 27280744 PMC5148735

[47] SOL Sociocultural Ancillary Study. Study procedures manual. 2010 Jan 12. (cited 2019 Apr 19). Available from: https://biolincc.nhlbi.nih.gov/studies/hchssol/

[48] Antoni MH, Lechner SC, Kazi A, Wimberly SR, Sifre T, Urcuyo KR, et al How stress management improves quality of life after treatment for breast cancer. J Consult Clin Psychol. 2006;74:1143–52. 10.1037/0022-006X.74.6.1152 17154743 PMC5752106

[49] Anderson EE. CIRTification: training in human research protections for community-engaged research partners. Prog Community Health Partnersh. 2015;9:283–8. 10.1353/cpr.2015.0044 26412769 PMC4711932

[50] De las Nueces D, Hacker K, DiGirolamo A, Hicks LS. A systematic review of community-based participatory research to enhance clinical trials in racial and ethnic minority groups. Health Serv Res. 2012;47:1363–86. 10.1111/j.1475-6773.2012.01386.x 22353031 PMC3418827

